# Recruitment process for studying occupational and environmental chemical exposures in a birth cohort study in Ecuador: challenges dealing with a stratified sample

**DOI:** 10.1186/s12884-026-09067-4

**Published:** 2026-04-23

**Authors:** Fadya Orozco, Stephanie Montenegro, Fabián Muñoz, Alexis J. Handal

**Affiliations:** 1 Centro de Transferencia de Tecnología, Universidad San Francisco de Quito USFQ, Quito, Ecuador; 2 Visor Análisis Estadístico Cía. Ltda., Quito, Ecuador; 3 Department of Epidemiology, University of Michigan School of Public Health, Ann Arbor, MI USA

**Keywords:** Environmental epidemiology, Community-engaged research, Birth cohort, Recruitment, Commercial Floriculture, Latin America

## Abstract

**Background:**

The Study of Environmental Exposure of Mothers and Infants Impacted by Large-Scale Agriculture (SEMILLA) is a community-based birth cohort study carried out in the Cayambe, Pedro Moncayo region, Ecuador, with intensive industrial floricultural activity. Maternal occupational and environmental pesticide exposure was assessed among three groups of pregnant women stratified according to "maternal occupational activity" at the time of recruitment. This article describes the recruitment process and strategies used, determines their effectiveness in targeting pregnant women with different levels of exposure, and discusses reasons that influenced recruitment rates.

**Methods:**

The projected sample was stratified into three occupational activity groups: floricultural/agricultural workers, non-agricultural workers, and non-workers (women not working outside the home for pay). Recruitment strategies include community activities, collaboration with health care providers, information from obstetric records (“Obstetric Census”), and non-monetary recognition. Analyses were conducted by occupational activity at the time of recruitment. Recruitment rates were calculated by occupational group. Monitoring indicators included the number and percentage of participants recruited by strategy. Wilcoxon rank-sum test and Fisher's exact test were used to compare characteristics between eligible and participating pregnant women; the Kruskal–Wallis test was used to compare among participant groups.

**Results:**

A total of 409 participants were recruited, 74% of floricultural/agricultural workers, 81% of non-agricultural workers, and 64.5% of non-workers. The strategy that referred the highest number of participants was the “non-monetary recognition” (60.9%), proving its effectiveness for recruiting floricultural/agricultural and non-agricultural workers (74.5% and 67.7%, respectively). The “Obstetric Census” was the second most important strategy (30.6%), especially effective in recruiting non-workers (49.6%). Compared to floricultural/agricultural and non-agricultural workers, non-workers were younger (*p* < 0.001) and had a significantly higher average number of prenatal check-ups (*p* = 0.0006). Floricultural/agricultural workers had a significantly higher average number of pregnancies and living children than the other two groups (*p* > 0.01). Time restrictions among women working outside the home for pay, and lack of autonomy among non-workers, were crucial factors that limited participation in the study.

**Conclusions:**

Stratifying recruitment by comparability groups requires differentiated strategies. Understanding living conditions and the social context of the study region are critical to effectively targeting recruitment efforts.

## Background

During pregnancy, occupational and environmental exposure to chemical contaminants at critical periods of susceptibility has lifetime and intergenerational effects on the fetus and infant [1]. In areas dedicated to industrial agricultural activities, both the agricultural and non-agricultural population have a high probability of being exposed to pesticides [2, 3]. In the case of women living in regions where there is intensive agricultural activity, they may be exposed to pesticides if they are employed in the commercial agricultural sector, are self-employed or small-scale farmers, their partner or household members work in the agricultural sector, or they live close to agricultural fields or farms; in this case, exposure may occur through various non-occupational pathways including water, dust, or food, which may be contaminated with pesticide residues resulting from application [3–5]. Globally, flower cultivation is one of the agricultural industries with the highest employment of female labor, 60–80% of workers are women, and is also one of the commercial industries with high levels of environmental contamination [6, 7]. In Ecuador, previous studies [8] identified the presence of pesticides (organophosphates, organochlorines and carbamates) in 67% of water samples from rivers and streams that received effluents from the flower industry. The country is the second largest exporter of flowers in the world; this industry is among the top five non-oil exports in the country [9, 10]. Moreover, the Ecuadorian flower industry is no exception to the global flower workforce employing a large proportion of female workers, with most of reproductive age.

In the case of community-based epidemiological cohort studies that examine the effects of occupational and environmental exposures on pregnant women and infants, participant recruitment poses several challenges. These include issues related to (1) the study’s hypothesis focusing on exposures during pregnancy and (2) to social and sociocultural factors, especially when the study involves populations from disadvantaged socioeconomic contexts [11]. Regarding the former, a distinctive feature of recruiting pregnant women for a birth cohort study is the rigorous eligibility criteria, including gestational age. Early recruitment is therefore essential, ideally during the first trimester of pregnancy or during the first 20 weeks of gestation at the latest, in order to examine critical windows of exposure during fetal and infant development. Moreover, these studies often report high rates of miscarriage, which further complicates the recruitment and enrollment process [12–14]. Under such conditions, recruitment becomes demanding in terms of time, resources and logistics, and requiring the implementation of multiple strategies to foster trust and acceptance among pregnant women and their social networks [11, 14]. As a result, the enrollment rates reported in the literature for this type of study range between 35 and 50% [13–16].

In birth cohort studies focused on the analysis of occupational and environmental exposures, stratifying recruitment across maternal occupational activities allows for greater comparability. This article describes the strategies developed to recruit pregnant women exposed to pesticide contamination in intensive commercial agriculture, mainly floriculture. The recruitment was conducted as part of the Study of Environmental Exposure of Mothers and Infants Impacted by Large-Scale Agriculture (SEMILLA) birth cohort, whose primary purpose is to examine the association between prenatal occupational and environmental pesticide exposure and child growth and neurocognitive development outcomes. The purpose of this manuscript is to describe the overall recruitment process and strategies used, assess their effectiveness in reaching pregnant women with different levels of pesticide exposure, and discuss the reasons that influenced recruitment rates in the Cayambe-Pedro Moncayo region in Ecuador. This manuscript focuses specifically on the recruitment phase of the SEMILLA study. The design, validity, and reliability of the exposure and outcome measures are addressed in detail in separate publications [17, 18]

## Methods

### Study setting and design

Implemented from 2018 to 2024, the SEMILLA project was designed as a community-based cohort study in the Cayambe-Pedro Moncayo area of Ecuador, a region known for intensive floricultural production. Led by researchers from Universidad San Francisco de Quito (FO) and the University of Michigan (AJH), the study examines the association between occupational and environmental exposure to pesticides, particularly the fungicide Mancozeb, a dithiocarbamate compound, and its main metabolite, ethylenethiourea (ETU). SEMILLA focuses on how these exposures, in relation to maternal occupational activities, affect the growth and neurocognitive development of infants and young children [17].

**Fig. 1:**
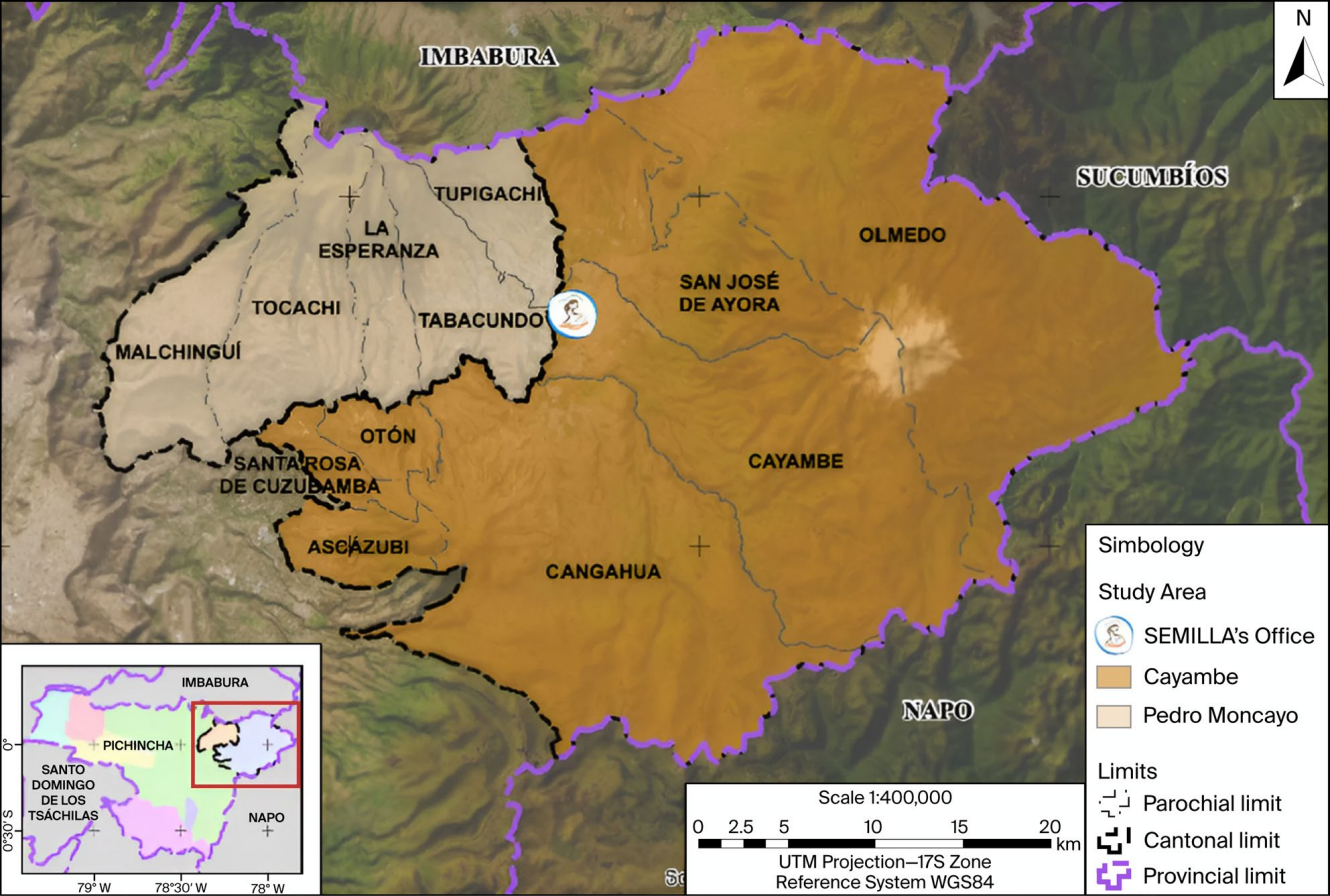
Study area of SEMILLA study [17]. Figure took with permission from: Handal A, Orozco F, Montenegro S, Cadena N, Muñoz F, del Rio ER, Kaciroti N. The Study of Environmental Exposure of Mothers and Infants Impacted by Large-Scale Agriculture (SEMILLA): Description of the Aims and Methods of a Community-Based Birth Cohort Study [17]

The study area is located northeast of Quito, the capital of the country, in the province of Pichincha, at a distance of approximately 90 km along a major highway (see Fig. 1). This region was selected because it concentrates 41% of the country's flower production [10], with a 60% female labor force [19], which is why it was the subject of previous studies that helped to situate the study hypothesis [4, 12, 20]. Geographically, the area includes the cantons of Cayambe and Pedro Moncayo, with a combined total area of 1,514 km^2^, the former being the largest (1,182 km^2^). The total population is approximately 149.062 inhabitants [21, 22], which are mainly concentrated in Cayambe canton (72%), where 54.5% reside in rural areas [21]. In Pedro Moncayo, the percentage of rural inhabitants is higher (70%%) [22]. As of 2017, due to the proximity of the region to the border crossings with Colombia, the migratory flow of people between 26 and 40 years of age, especially from Venezuela and Cuba, who choose to reside especially in urban areas in the region, has increased compared to previous years [23].

Ethical approval for the study was granted by the Human Research Ethics Committee of the Universidad San Francisco de Quito (CEISH ID 2017-177IN), and by the Health Sciences and Behavioral Sciences Institutional Review Board of the University of Michigan (HUM00138211). Additionally, the District Health Directorate 17D10 Cayambe Pedro Moncayo, of the Ecuadorian Ministry of Public Health (MSP, *Ministerio de Salud Pública*), authorized the implementation of the study in the area.

Pregnant women were recruited between 8 and 20 weeks of gestation. The initial projected sample was 420 participants, stratified by according to the category "maternal occupational activity" at the time of recruitment into three groups: (1) floricultural/agricultural workers, (2) non-agricultural workers, both groups composed of women working outside the home for pay; and (3) non-workers, defined as women not engaged in paid employment outside of the home, based on findings from a previous study [20]. These three groups were key to address SEMILLA study aims, allowing for comparability [17]. Given that recruitment was ongoing during the pandemic and post-pandemic COVID-19 period and considering the increasing levels of labor inactivity and female unemployment in the agricultural sector in general [24], many pregnant women were unemployment at the time of enrollment. Furthermore, though the focus of the study was on floricultural workers, given the shift of these workers to other agricultural industries during the pandemic, when the flower industry was heavily impacted [25], floricultural and other agricultural workers were included in the first occupational group. Inclusion criteria were defined based on previous studies conducted in the same population [4, 12, 20]. Participants were eligible if they met all of the following conditions: (1) were 18 years of age or older; (2) had lived in the study area (Cayambe or Pedro Moncayo cantons) for at least one year prior to recruitment; and (3) expressed the intention to remain in the study area for at least one year after delivery. Women under 18 years of age were excluded due to national ethical regulations requiring parental consent for minors. This requirement posed potential concerns regarding the confidentiality of pregnancy status, which is particularly sensitive in this population.

Participation in the study required an investment of two to three hours every three months during pregnancy (approximately once per trimester) and thereafter until the baby reached 18 months of age, with a single visit thereafter between 24 and 39 months for a subset of the study population who qualified based on their initial enrollment date. Detailed descriptions of the study protocol are described elsewhere [17]. Briefly, during pregnancy, at each visit, the participant was asked to provide 10 ml of blood, approximately 20 ml of urine, a hair sample, and a toenail sample from both feet. In addition, a questionnaire was administered to assess sociodemographic characteristics, recent occupational history, occupational and environmental chemical exposures, lifestyle habits, psychosocial stressors, and several cognitive and psychometric scales (e.g., depression, anxiety). At the time of birth, a questionnaire was administered to the mother and at approximated 1–2 weeks post-partum, a venous blood sample was collected from the baby for thyroid hormone analysis.

During postnatal follow-up visits, mothers completed several of the same questionnaires administered during the prenatal period, including those related to exposure and occupational history, nutritional status and some psychosocial scales. Infants were evaluated every three months and underwent nutritional and anthropometric assessments, neurodevelopmental evaluations (including age-appropriate cognitive and motor scales), and capillary blood sampling to assess hemoglobin levels (via HemoCue). Mothers were informed during recruitment about the postnatal follow-up schedule and the specific types of evaluations that would be conducted with their child. A detailed study timeline is available in Handal et al. [17]. Participation in postnatal assessments was voluntary and covered under the informed consent process.

### Recruitment process

**Fig. 2:**
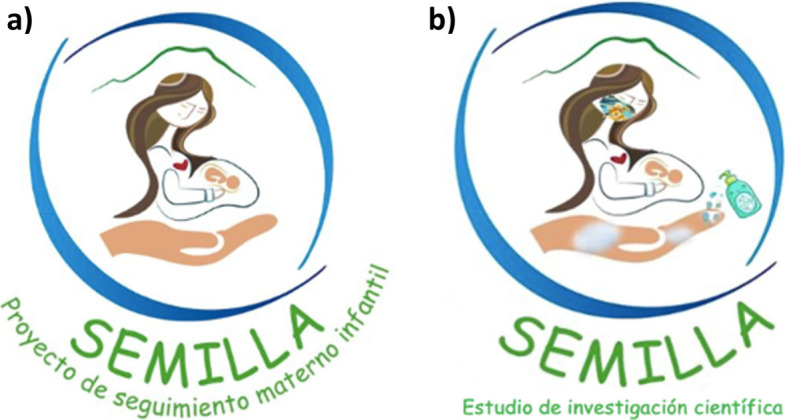
SEMILLA study logo. **a** Study logo used during the study implementation. **b** Study logo using specifically during COVID-19 Pandemic period

The recruitment process took place over a period of 30 months, from October 2019 to April 2022, with the COVID-19 pandemic and major social protests in the country that forced the shutdown of field activities at variable intervals during the years 2019, 2021 and 2022 [26–28]. Before recruitment began, the team assessed the area’s social context, which involved dissemination of the study among public and private health care providers, strategic actors and key stakeholders, and the general public, with the objective to gain support and foster confidence to enhance expectations for participant recruitment and engagement. As part of the project's social insertion activities, a logo was designed to position the study in the context of the population, valuing aspects such as the mixed race of Cayambe and Pedro Moncayo women (dark brown hair and brown eyes), and the silhouette of the Cayambe volcano, geographical symbol of the area, resting on a hand to represent SEMILLA's support to the participating mothers during their pregnancy. The logo was adapted for use before and during the COVID-19 pandemic (see Fig. 2).

The field recruitment team consisted of three people in charge of the first contact with potential participants, internally called "recruiters." These were women from the area, with technical and university training in health-related disciplines, experience working in the field, and intercultural sensitivity and flexibility regarding the time constraints of the pregnant women. The team was trained by the Technical Coordinator of the SEMILLA project (SM) on the main objectives of the study and the inclusion and exclusion criteria. Prior to the start of field activities, the recruiters underwent human subject’s protection training and obtained the standard "CITI Program" certificate [29], which guarantees their knowledge of research ethics and compliance and confidentiality of information. The Technical Coordinator oversaw supervising the team of recruiters, as well as coordinating with health care providers in the area the referral of potential participants, who were then approached by the recruiters to verify the inclusion criteria and their willingness to participate in the study.

The recruitment process began with the referral of a potential participant. In the case of health care providers who served as referrers, they asked the pregnant woman for her verbal consent to be approached at a later time via telephone by SEMILLA staff. In this phase, the pregnant mothers, called *referred*, were approached by one of the recruiters to verify contact information. Those women who answered the telephone call were designated as *contacted*. The purpose of the study was briefly explained to them asking whether or not they were interested in participating, and sociodemographic information was requested. In the case of women who did not answer their telephone, several attempts were made in the following days. Those who completed this phase and stated that they were interested in participating were called *recruited.* Subsequently, the team verified whether the recruited were eligible, confirming the criteria for inclusion in the study. Those who met all the criteria were referred to as *eligible*. Finally, this group had their gestational age confirmed by means of an ultrasound, subsidized by the project and performed by a local health care provider with whom the study had an agreement of collaboration. The prenatal ultrasound performed at enrollment was used solely to verify gestational age for study eligibility and scheduling of follow-up visits. It was not intended as a diagnostic examination, and participants were informed that any findings requiring further evaluation would be referred to their usual health care provider.

In addition, an appointment was scheduled with the Technical Coordinator, who reiterated the purpose of the study and provided time for the interested women to ask any questions they might have. The women could be accompanied by their partner or a family member if they wished. At the end of this meeting, which lasted approximately one hour, their consent to participate in the study was requested. Thus, expectant mothers who agreed to sign the informed consent form were referred to as *participants*. Informed consent was obtained in writing and a copy was provided for reference. Whenever possible, eligible pregnant women who decided not to participate in the study were contacted by telephone, or a visit was made to their homes with their prior knowledge and authorization to try to understand the reason for their decision not to participate.

During the initial telephone contact and any subsequent recruitment interactions, the team followed a structured script that included verifying basic eligibility criteria (e.g., age, residence duration, gestational age), collecting contact and sociodemographic information (e.g. employment status), and briefly explaining the objectives and procedures of the SEMILLA study. This script served as a guide to ensure consistency in the information provided and collected, although it did not constitute a formal questionnaire. All interactions were conducted in Spanish, with culturally sensitive language, and adapted to the time availability of the women. Detailed study procedures, including the full data collection instruments, are described in the SEMILLA protocol [17].

### Non-monetary participant acknowledgment

As part of the study design, modest non-monetary tokens of appreciation were provided to participants at key points in the study to recognize their time and engagement, and to help reduce logistical barriers to continued participation. At enrollment, *participants* received a small food basket containing nutritious items such as fresh fruits, vegetables, and local dry products (e.g., lentils and cereals). This gesture was intended to acknowledge their time spent during study visit and support household nutrition. At the time of delivery, a small package of baby clothes was provided, while at each infant follow-up visit, an educational toy appropriate for the baby's age was provided (e.g., a ball for motor development, a star to place shapes accordingly, a tower of stackable rings, etc.). In addition, baby hygiene and nutrition items, such as training cup, toothbrush, and toothpaste, were offered to encourage healthy habits.

Throughout follow-up, participants also received health-related information and feedback on biological tests results when abnormal values were detected, along with support in referral to public health services when necessary. Mothers were provided with general guidance on nutrition and infant stimulation. In some cases, participants received emotional support from a trained member when mental health needs were identified, particularly during the post-pandemic period.

During and after the COVID-19 pandemic, the study supported vulnerable pregnant women with additional food baskets, either because they or their partners had lost their jobs, To address participant’s mobility limitations, many of whom lived in remote rural areas or faced long travel times (up to 90 min each way), along with unfavorable economic conditions and the need to comply with biosecurity measures, the SEMILLA study covered the cost of private transportation from each participant’s home to the study office.

These modest acknowledgments were designed in accordance with ethical standards approved by the Institutional Review Boards and were not intended to exert any undue influence on participants’ decision to enroll or remain in the study.

### Recruitment strategies

The outreach and recruitment strategies were diverse, and included community activities, cooperation with health professionals in the area, other key stakeholders, and promotion of the study through different means of communication. Collaboration with Ministry of Health personnel at the first level of care in the area was strategic in order to disseminate the study through its different spaces, including fifteen health care facilities (nine primary health care centers in Cayambe and five in Pedro Moncayo) and a public Basic Hospital in Cayambe, in addition to off-site activities.

The main strategies used are presented below in the order in which they were implemented. Due to the stratification of the sample according to the category "maternal occupational activity", it was necessary to devise new ways of finding and recruiting interested pregnant women who met the inclusion criteria and had availability to participate. This last aspect was especially important in the recruitment of pregnant women working in floricultural/agricultural activities who often work long hours with limited free time.

#### Community meetings

To spark interest in the study, the study field team, made up of the recruiters and the Technical Coordinator, visited several rural communities in the area that had the highest proportion of women of childbearing age, relative to the total community population, based on census and health information available for the region. During a period of ten minutes, the study field team explained the objective, inclusion criteria, and the benefits of participation, including non-monetary forms of recognition. The visits took advantage of regularly scheduled community meetings that community members are required to attend or otherwise face fines for non-attendance, which are scheduled by community leaders to discuss matters of public interest. These meetings generally took place early in the morning (03:00) or in the evening (19:00) and were well attended by community members, as participation was considered mandatory by local leaders and community organizations as part of community governance practices. These visits were facilitated by front-line Ministry of Public Health personnel, called Primary Health Care Technicians (TAPS, *Técnicos de Atención Primaria en Salud*).

#### Home visits in close collaboration with public health care providers

Home visits were guided by the TAPS, during which the field team visited the homes of pregnant women, following the planning established by the MSP as part of its off-site activities. The link with the TAPS made the people who were visited more open to listening to the information about the study. At the end of each visit, a card with SEMILLA's contact information was handed out, so that those who were interested could communicate or, if necessary, refer others. The main limitation of this strategy was the time and cost of travel, especially to more remote rural areas, limiting the number of pregnant women the team could approach. Because each Primary Health Care Technician (TAPS) was responsible for areas that varied in size, population density, and distance between communities, the number of women visited fluctuated between two and three at a time.

#### Television interviews

Interviews were scheduled on the main local television channels. The Technical Coordinator provided information about the study and its benefits for the community, including recognition for participant engagement and contact information. Additionally, each channel rebroadcast these interviews on Facebook, the main social network used in the study area, fostering greater interest.

#### Posters

A printed poster describing the main inclusion criteria and SEMILLA's contact number was developed, inviting pregnant women to participate. The posters were placed in different places, such as health clinics, clinical laboratories, pharmacies, and places where people gathered, such as bus stops and food stores and supermarkets. At the same time, general information about the study was provided to the owners or those in charge of customer service in those places.

#### Radio advertisements

Based on previous experience from research studies conducted in the area [20], radio announcements were used as a strategy. The main radio station in the area was chosen for this purpose. The advertisement was developed in consultation with the radio station. A woman's voice was used to warmly and enthusiastically invite the pregnant population to participate in SEMILLA. Additionally, the advertisement mentioned the inclusion criteria, the support and benefits participants would receive, and a contact number. The transmission times of the radio announcement were selected according to the audience, as recommended by the radio station, in the morning between 06:00 and 07:00, and in the evening at 18:00, skipping one day, over 3 months.

#### Facebook fan page

For this strategy, a fan page was created on the social network Facebook, which is widely used and known by the population in the study area. Initially, a digital poster was published inviting people to participate in SEMILLA, emphasizing the inclusion criteria, and inviting those interested to request information via the page's chat. Subsequently, to make the page more attractive and promote people's interest in its content, specialized topics related to pregnancy, breastfeeding, baby development and growth, responsible parenting and parenting models were addressed. Some of these topics were presented in the form of infographics, while others were addressed through conferences with specialized professionals. The purpose was that the pregnant women or their families would find the information presented useful and would contact the field team requesting more information that would lead them to participate in the study. The fan page was especially useful to maintain connection between SEMILLA and the local population during the COVID-19 pandemic.

All formal recruitment materials, including printed posters and radio scripts, were reviewed and approved by the Institutional Review Boards prior to their dissemination. Informal outreach efforts via social media were based on IRB-approved language but were not individually submitted for review.

#### Visits to public health care clinics

Regarding maternal services, the Ecuadorian government guarantees prenatal care for all women through the Ministry of Public Health (MSP). Those who are formally employed also have the coverage of the Ecuadorian Social Security Institute (IESS, *Instituto Ecuatoriano de Seguridad Social*). Alternatively, current legislation states that all companies with 100 or more employees must offer a basic health service in their facilities through an occupational physician, as is the case of many flower companies in the area.

This strategy was developed exclusively in the MSP facilities, and focused on the recruiters' periodic visits to the obstetric consultation waiting rooms in all the health care facilities of the MSP in the area, inviting the pregnant women who were there to participate. The recruiters presented the study and explained its voluntary nature, along with general information about what participation would involve. They also mentioned the types of modest non-monetary tokens of appreciation that participants and their babies would receive throughout the study. When appropriate, examples of these items, such as a small food basket or baby care supplies, were shown to ensure transparency and help participants make informed decisions.

Health personnel, mainly midwives and physicians from the different health care facilities, did not participate directly in the recruitment activities. Instead, they were much more active in referring potential participants through the institutional spokesperson strategy, which will be mentioned later.

Unfortunately, it was not possible to develop this strategy in the health care facilities under the administrative structure of the IESS. Aspects such as high centralization and low levels of administrative oversight at the local/regional levels made it challenging for IESS to collaborate. Despite repeated visits by one of the study's principal investigators (FO) and the study's Technical Coordinator to try to gain support for the study and access to local health providers in the area, the participation of this actor was not possible. Furthermore, neither the recruiters nor the Technical Coordinator were given access to the health services facilities of the companies present in the area, floriculture and others, for the socialization of the study among their health personnel, due to institutional restrictions mentioned by these players.

#### Obstetric records “obstetric census”

Public healthcare facilities use the “Obstetric Census”, an Excel spreadsheet, to record pregnant women's catchment population under their purview. During and after the COVID-19 pandemic, the dissemination activities of the study in the social context of the Cayambe-Pedro Moncayo area were limited. In order to continue recruiting, the midwives of the MSP provided information to the study by sending the spreadsheets. These records contained basic demographic contact information of the pregnant woman, such as name, telephone number, and weeks of gestation, among others. Unfortunately, the compilation of these records was delayed, so that many of the pregnant women referred to at the time they were contacted no longer met the required gestational age. In addition, there were errors in the contact data (for example, invalid telephone numbers) so that much of the information was not accurate. Despite these challenges, these records made it possible to locate the areas with the greatest number of pregnant women, which was used to guide recruitment with other strategies, including the promotion of the study with possible community spokespersons. After their use, and for reasons of confidentiality, the spreadsheet dataset was eliminated from all study computers.

#### Active community level outreach

One of the recruiters went to places with large concentrations of people, such as transportation stops of floriculture companies, fairs, markets, and others, in order to promote the research project. For this purpose, the recruiter had a five-minute speech prepared, and audio-visual material explaining the activities that were carried out during the participation in SEMILLA. In addition, there were video testimonials from other participants, and some of their partners, recorded with their consent, who invited them to be part of the study. The recruiter also showed examples of the non-monetary recognition items provided to participants.

#### Non-monetary recognition through institutional and community spokespersons

To promote and strengthen collaboration with health personnel and community members in the recruitment process, a modest non-monetary recognition strategy was implemented. As part of this approach, basic contact details of potential participants were recorded on a physical referral card, which could later be exchanged for tokens of appreciation, such as vouchers for food, personal hygiene items, or other everyday goods. Data gathered included: gestational age, verbal agreement to be contacted by the SEMILLA team for further information, and contact telephone number. Subsequently, the physical card was replaced by a WhatsApp message sent to one of the recruiters, as this method proved more accessible for both health personnel and community members, particularly given time constraints. People who referred potential participants through this strategy were referred to as *spokespersons*. These spokespersons were classified into two groups: *institutional* and *community*. The *institutional spokespersons* were mainly public and private health care providers in the area who, due to their role and functions, had frequent contact with pregnant women, most notably MSP midwives and the physician who performed the study’s ultrasound scans. The *community spokespersons* were people from the community, participants, family members of participants, and various service providers with whom the field team worked closely (private drivers, fruit vendors, office supply providers, among others).

In order to keep track of the acknowledgments provided, a registration system was used to count referrals, and on a monthly basis, the spokespersons with the highest number of referrals received a small additional token of appreciation. This approach helped to maintain engagement and foster collaboration among health personnel and community members in identifying eligible participants.

#### Direct communication

Potential participants could communicate directly with the SEMILLA field team, using the study contact information, based on the recommendations of their acquaintances or health care providers.

### Tracking of the recruitment process

Due to the stratification of the sample according to the category "maternal occupational activity", a rigorous follow-up system was implemented to optimize resources for the recruitment of pregnant women according to the referral source. Initially, when the pregnant woman was in the contact stage, basic information such as name, age in years, gestational age, interest in participating, referral source, confirmation of contact telephone number was recorded in the database. Subsequently, when the pregnant woman was recruited, she was asked about her employment status (work for an economic income: YES/NO), and occupational activity (floricultural/agricultural, non-agricultural, and non-worker). In this regard, occupational activity information was preliminary, to be finalized and confirmed in the baseline questionnaire administered upon official enrollment into the study.

In addition, when the pregnant women were considered eligible, they were asked about their marital status, years of education, number of hours of paid work per week, number of pregnancies, number of prenatal check-ups, number of living children, and number of abortions. Pregnancy loss was asked about because previous research in the area [12] reported a 3.4 times higher probability of miscarriage (CI: 95% 1.3–8.8) in women working in the flower sector compared to those working in another sector. In these cases, telephone and sometimes home follow-up was more frequent. Like the maternal occupational activity data, sociodemographic and reproductive health information was preliminary, to be finalized in the baseline questionnaire upon enrollment.

The recruitment target was approximately thirty-five women per month, especially in the period after the COVID 19 pandemic.

### Other recruitment considerations

In addition to the aspects related to the COVID-19 pandemic and social protests in the country, there were factors related to the dynamics of the socio-cultural context of the area, which influenced the pregnant women's decision of whether to participate in the study. The first of these has to do with the effects of *machismo* on the decisions and autonomy of women, both married and single, by husbands, fathers, and brothers, who in many cases ended up deciding whether or not the pregnant woman could participate or continue in the study. In this regard, in a previous analysis carried out in the area, Newman and collaborators (2001) [19] report jealousy as one of the main causes of intra-family violence that limits women's freedom in terms of mobility. These same authors mention that although the rise of women's participation in floriculture work has improved the balance of power in gender relations, it has nevertheless been a source of family conflicts, especially around reproductive activities, as they are unable to delegate or demand that their partners take care of their children. These aspects should be contrasted with the time of data collection during the follow-up period. On the other hand, prior to the start of the study, some cases of "baby stealing" had been reported in the local press in the area [30], so the pregnant women and their families were reluctant to be contacted by the study, despite being referred by a health care provider. For this reason, the positioning of the study in the social context was a necessary strategy for the study, in terms of generating trust in the population through different channels.

### Statistical analysis

Recruitment rates were calculated by dividing the number of participants enrolled in the study by the number of eligible women overall and according to maternal occupation activity. Information about recruitment strategies and process was registered using the Excel software. The effectiveness indicators used to monitor recruitment were: 1) number and percentage of total pregnant women referred by strategy; 2) number and percentage of pregnant women referred by strategy, according to "maternal occupational activity" (floricultural/agricultural, non-agricultural, and non-workers); and 3) number and percentage of study participants by strategy according to "maternal occupational activity" effectively recruited.

Comparing sociodemographic and reproductive health characteristics of *eligibles* and *participants*, and between *participants* according to stratification groups by occupational activity, enhanced understanding of the factors influencing the recruitment rates in each category. Wilcoxon rank-sum test was used for the comparison between medians, and Fisher's exact test was used for the case of nominal variables. The Kruskal–Wallis nonparametric test was used to compare participants according to groups by maternal occupational activity.

Analyses were performed using the SAS statistical package, Version 9.1.

## Results

### Recruitment rates

**Fig. 3:**
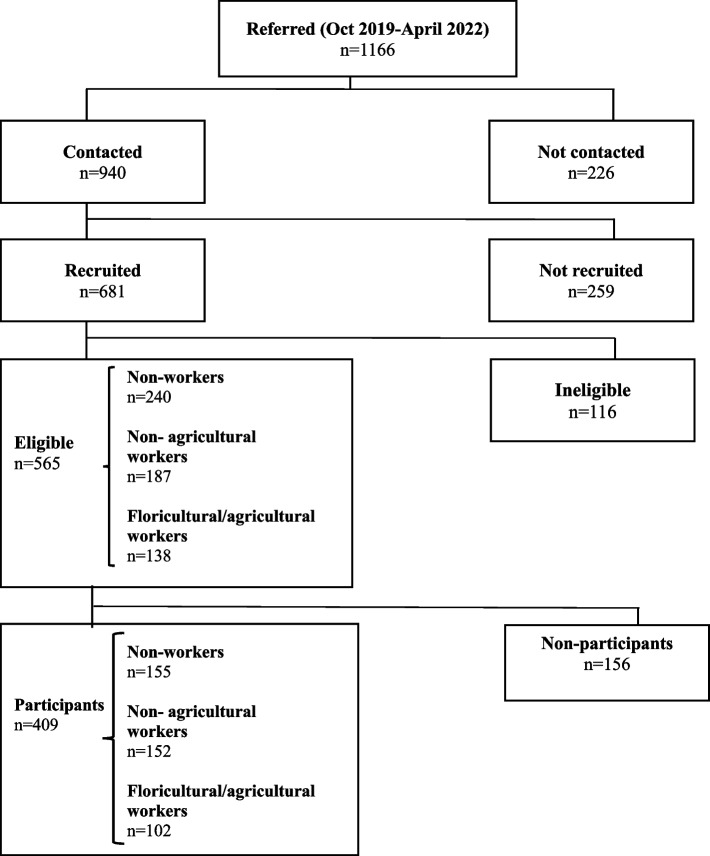
Flowchart of the recruitment process. SEMILLA study, Cayambe, Ecuador, 2018–2024

Through the aforementioned strategies, 1,166 pregnant women were *referred*, of which 940 (80.6%) were *contacted*. Due to incorrect contact numbers, 226 referred pregnant women could not be reached (see Fig. 3). Of the 681 pregnant women in the *recruited* group, 83% (*n* = 565) were eligible. Finally, the total sample consisted of 409 *participants*, with an overall recruitment rate of 72.4%. Among the three groups defined by maternal occupational activity, the recruitment rate was 73.9% (102/138) among floricultural/agricultural workers, 81.3% (152/187) among non-agricultural workers, and 64.5% (155/240) among non-workers.

There were several reasons why 27.6% of the *eligible pregnant women did not participate* in the study (*n* = 156). The main reasons were as follows: 1) Lack of time (6.4%), with 80% of these cases corresponding to floricultural/agricultural and non-agricultural workers. 2) Reproductive health complications such as spontaneous abortions and anembryonic pregnancies (4.5%), of which 86% corresponded to non-agricultural workers (*n* = 3), and women non-workers (*n* = 3). 3) Loss of contact (52%), most frequently among non-workers (60.1%). 4) Decision not to participate, without any explanation (22.4%), of whom 57% were non-workers. 5) Explicit mention of not having permission from partners or family members to participate (9%), with 43% of these being non-workers. 6) Not meeting the selection criteria at the time of enrollment, mainly gestational age confirmed by ultrasound or change of residence outside the study area (5.8%), of which 55.5% were non-workers (*n* = 5) (data not shown in tables).

### Effectiveness of recruitment strategies

In general, the most effective recruitment strategies for the total referral of pregnant women were the “Obstetric Census” (46%) and the “non-monetary recognition” (44.6%). Strategies such as direct communication and community outreach were less effective (3.8% and 3% respectively), as was the Facebook fan page (2.4%). Other strategies such as radio and posters were ineffective (0.1% in each case) (see Table 1). Strategies like community meetings, home visits, television interviews and direct contact through clinics were not effective.

**Table 1:** Effectiveness of recruitment strategies for pregnant women by maternal occupational activity: SEMILLA study, Cayambe, Ecuador^*^

Strategy	Referred, n (%)	Participants, n (%)	Floricultural/Agricultural workers, n (%)		Non-agricultural workers, n (%)		Non-workers, n (%)	
			Referred	Participants	Referred	Participants	Referred	Participants
Obstetric Census	536 (46)	125 (30.6)	44 (26.2)	18 (17.6)	53 (24.1)	30 (19.7)	211 (52.8)	77 (49.7)
Non-monetary Recognition:								
Institutional Spokesperson	314 (27)	145 (35.5)	68 (40.5)	42 (41.2)	89 (40.5)	65 (42.7)	86 (21.5)	37 (23.9)
Community Spokesperson	205 (17.6)	104 (25.4)	42 (25)	34 (33.3)	55 (25)	38 (25)	71 (17.8)	33 (21.3)
Active Community Level Outreach	34 (3)	14 (3.4)	4 (2.4)	3 (2.9)	11 (5)	9 (6)	8 (2)	2 (1.3)
Direct Communication	45 (3.8)	13 (3.2)	6 (3.6)	2 (2)	8 (3.6)	6 (4)	17 (4.3)	5 (3.2)
Facebook Fan Page	28 (2.4)	4 (0.9)	3 (1.8)	2 (2)	1 (0.4)	1 (0.6)	6 (1.5)	1 (0.6)
Posters	2 (0.1)	2 (0.5)	0	0	2 (0.9)	2 (1.3)	0	0
Radio	2 (0.1)	2 (0.5)	1 (0.6)	1 (1)	1 (0.4)	1 (0.6)	0	0
Total	1166	409	168	102	220	152	399	155

^*^The table does not include 379 referred women who could not be classified by maternal occupational activity at the time of contact or recruitment

Regarding the number of pregnant women referred by strategy, according to “maternal occupational activity" (floricultural/agricultural, non-agricultural, and non-workers), within the “non-monetary recognition”, institutional spokespersons were an effective means for the referral of women in the maternal occupational activity of floriculture/agriculture and non-agriculture (40.5% in each group). In the case of non-workers, the most effective referral strategy was the “Obstetric Census” (52.8%) (see Table 1).

Finally, the strategy that referred the highest number of participants was the “non-monetary recognition” (60.9%), with the referral of institutional spokespersons being very effective (35.5%). This strategy made it possible to recruit the highest percentage of pregnant women in the three occupational activities, being the most effective for floricultural/agricultural and non-agricultural workers (74.5% and 67.7%, respectively). The “Obstetric Census” was the second most important strategy for recruiting participants (30.6%), being especially effective in recruiting non-worker pregnant women (49.6%) (see Table 1).

### Sociodemographic and reproductive health characteristics of ‘eligibles’ and ‘participants’

From the total number of *participants*, 102 participants were floricultural/agricultural workers, 152 were non-agricultural workers, and 155 were non-workers, at the time of recruitment. In general, there was no statistical differences between the population of *eligible* pregnant women and *participants* in any of the three groups by maternal occupational activity (*p* > 0.05), according to sociodemographic and reproductive health characteristics (see Table 2).

**Table 2:** Sociodemographic and reproductive characteristics of eligible vs. participating pregnant women by occupational activity: SEMILLA study, Cayambe, Ecuador

Characteristics	Floricultural/Agricultural workers			Non-agricultural workers			Non-workers		*P* value^a^
	Eligible (*N* = 138)	Participants (*N* = 102)	Eligible (*N* = 187)	Participants (*N* = 152)		Eligible (*N* = 240)	Participants (*N* = 155)		
Age									< 0.001
Mean (SD)^1^	28.7 (5.5)	28.8 (5.5)^b^	27.7 (6.1)		27.6 (6.1)^b^	25.9 (5.7)		25.8 (5.5)^b^	
Median (Range)	28 (18–48)	28 (19–48)	27 (18–46)		27 (18–45)	25 (18–46)		25 (18–46)	
Years of education									< 0.001
Mean (SD)	11.1 (3.1)	11.1 (3.2)^b^	13.2 (3.6)		13.0 (3.7)^b^	12.0 (2.9)		12.0 (2.9)^b^	
Median (Range)	12 (4–18)	13 (4–18)	13 (4–18)		13 (4–18)	13 (4–18)		13 (4–18)	
Number of prenatal check-ups									0.0006
Mean (SD)	2.0 (1.3)	1.9 (1.3)^b^	2.1 (1.2)		2.0 (1.2)^b^	2.6 (1.5)		2.6 (1.6)^b^	
Median (Range)	2 (0–7)	2 (0–5)	2 (0–7)		2 (0–7)	2 (0–12)		2 (0–12)	
Number of pregnancies									0.0021
Mean (SD)	1.6 (1.1)	1.6 (1.1)^b^	1.2 (1.3)		1.3 (1.0)^b^	1.3 (1.2)		1.3 (1.2)	
Median (Range)	1 (0–6)	2 (0–5)	1 (0–7)		1 (0–7)	1 (0–6)		1 (0–6)	
Number of living children									0.0016
Mean (SD)	1.3 (0.9)	1.3 (0.9)^b^	0.9 (1.1)		0.9 (1.1)^b^	1.0 (1.0)		1.0 (1.0)^b^	
Median (Range)	1 (0–4)	1 (0–4)	1 (0–6)		1 (0–6)	1 (0–5)		1 (0–5)	
Number of abortions									0.085
Mean (SD)	0.3 (0.5)	0.3 (0.5)	0.2 (0.5)		0.2 (0.5)	0.3 (0.5)		0.3 (0.5)	
Median (Range)	0 (0–3)	0 (0–2)	0 (0–2)		0 (0–2)	0 (0–3)		0 (0–3)	
Number of hours of paid work per week						NA		NA	0.7092
Mean (SD)	40.3 (8.5)	39.5 (8.4)	40.4 (19.9)		41.3 (19.7)				
Median (Range)	40 (3–78)	40 (3–60)	40 (6–105)		40 (6–105)				
Place of residence, N (%)									0.035
Cayambe	87 (63)	66 (64.7)	143 (76.5)		119 (78.3)	185 (77)		119 (77)	
Tabacundo	51 (37)	36 (35.3)	44 (23.5)		33 (21.7)	55 (23)		36 (23)	
Marital status, N (%)									0.061
Married	32 (23.2)	24 (23.5)	56 (30)		46 (30.3)	50 (20.8)		32 (20.7)	
Free union/Common law	80 (58)	59 (57.8)	78 (41.7)		64 (42.1)	129 (53.8)		86 (55.5)	
Single/Divorced/Widowed	26 (18.8)	19 (18.6)	52 (27.8)		42 (27.6)	61 (25.4)		37 (23.8)	

^a^ *P* value reported the overall test among the three groups of participating pregnant women

^b^ Statistically significant differences among groups of participants at < 0.01

^1^ SD: Standard Deviation

When comparing participants across the three occupational groups, floricultural/agricultural workers had the highest average age (28.8 years), followed by non-agricultural workers (27.6 years), while non-workers were the youngest (25.8 years) (*p* < 0.001). In terms of years of education, floriculture/agriculture workers had, on average, fewer years of formal education (11.1 years), while non-agricultural workers had the highest average (13.0 years) (*p* < 0.001).

Most of the participants (average 73%) in the three groups resided in the Cayambe canton. Among floricultural/agricultural workers, 57.8% were in a common-law marriage, representing the highest proportion among the three occupational groups. Non-agricultural workers had the highest proportion of both married women (30.3%) and those who were single, widowed, or divorced (27.6%). Among non-workers, 55.5% were in a common-law union. Overall, more than 42% of participants in all occupational categories reported being in a common-law marriage. However, there were no statistically significant differences between groups in terms of these sociodemographic variables (*p* = 0.06) (see Table 2).

Regarding reproductive health characteristics, floricultural/agricultural and non-agricultural workers had fewer prenatal check-ups on average (2.0 and 1.2, respectively) compared to non-workers, who had the highest average (2.6) (*p* = 0.006). Regarding the number of previous pregnancies, participants working in floricultural/agricultural activities had a significantly (*p* = 0.0012) higher average number of pregnancies than non-agricultural workers (1.6 vs. 1.3, respectively). Floricultural/agricultural workers also had a significantly higher average number of living children compared to non-agricultural workers and non-workers (1.3 vs. 1.0 and 0.9, respectively; *p* = 0.016). There was no significant difference in the number of previous abortions among the three groups (*p* = 0.085).

Finally, regarding the average number of weekly working hours, there was no statistical difference (*p* = 0.709) between floricultural/agricultural and non-agricultural workers (39.5 h vs. 41.3 respectively).

## Discussion

Recruitment strategies included traditional community-based approaches, such as community meetings, home visits, active outreach, and the use of the media through posters, mass media, and social media (e.g., Facebook). Alternative and innovative strategies were also implemented, involving collaboration with health personnel from public and private health centers and clinics through direct referrals, with institutional and community spokespersons playing a key role in outreach and participant engagement. The support of public health care providers, particularly through the sharing of information from pregnant women’s records such as the Obstetric Census, was essential. Among all strategies used, those that were most effective for the *total referral* of pregnant women and participants were the Obstetric Census and the Non-monetary Recognition.

In the case of the Obstetric Census, three factors may have influenced its effectiveness as a recruitment strategy. The first has to do with a series of current regulations in Ecuador in place since 1994 that have increased prenatal care coverage, including free prenatal care [31]; thus, by 2012 it was reported that 79% of pregnant women had their first prenatal check-up within the first trimester of gestation [32]. The second factor may be related to the social dynamics of female labor during the COVID-19 pandemic and post-pandemic periods. During this time, the stratified recruitment of pregnant women, especially from the floricultural/agricultural and non-agricultural worker groups, was complicated by increasing levels of female labor inactivity and unemployment [24]. It is quite possible that these women, not being covered by the Social Security laws, were displaced to other providers of the Integral Public Health Network, among them mainly the Ministry of Health (MSP), which provides coverage to the population without registered affiliation or right to social security coverage. Finally, the third factor may be related to the geographic coverage and accessibility of public health services in the study area, given that in Cayambe-Pedro Moncayo, there is good urban and rural accessibility, especially by providers of the MSP. For these reasons, this strategy was especially important in the recruitment of non-worker participants (49.6%). On the other hand, the participation of midwives from the MSP public health services was strategic for the recruitment of pregnant women. In the recruitment process, midwives are key personnel in this type of study because of their close relationship with pregnant women, regardless of their level of obstetric risk. In addition, given that most of these personnel are located in geographically isolated or rural areas, they are the first contact for women in these localities [33]. In addition, they are the ones who handle and manage the collection of data of the health services under their responsibility, and they are often in charge of coordinating extramural activities and actively searching for pregnant women in the area or region they supervise [33].

Acknowledging the contributions of health care providers and community spokespersons through modest non-monetary recognition helped to increase the participation rate of pregnant women in the study. This aspect is important to generate an effective commitment from different actors in the recruitment process [13, 14, 31, 34]. The non-monetary recognition strategy was very effective for the recruitment of pregnant women in all occupational groups; however, it was decisive for the recruitment of participants from the floricultural/agricultural and non-agricultural occupational groups. In the case of the institutional spokespersons, the participation of one of these providers who offered ultrasound services for the confirmation of gestational age was a very important source of reference. Consistent with what has been observed in other studies, recruitment of women directly from ultrasound clinics results in response rates of 30–85% [35, 36]. It is possible that pregnant women who were working for pay, had less time to access public health care providers, went to private health care providers to have their pregnancy confirmed, or learned about the study through community contacts.

The overall enrollment rate of 72.4% was successful, compared to previous studies with women up to 20 weeks of gestation, where the rates ranged from 35 to 50% [13–16]. In community settings, the rejection rate is higher than when recruitment occurs in clinical settings, 32% versus 24% [37]. Galea and Tracy [38] state, in general terms, a sustained decrease in the interest of individuals to participate in studies of scientific interest over the last five decades. Among the factors related to recruitment, the fact that there are currently many telemarketing campaigns that confuse people, who therefore refuse to be contacted by telephone, is mentioned. In our case, this aspect could have influenced the initial rejection rate (19%) in the pregnant women who were not contacted. On the other hand, differentiated and common reasons were observed among the stratified groups of eligible pregnant women, related to the characteristics of their livelihoods that could have influenced their participation. Among these, lack of autonomy was more frequently mentioned by non-workers. These women were also younger than floricultural/agricultural and non-agricultural workers, which may have implied greater restrictions on their autonomy by their partners and family members, an issue noted in previous studies [35]. This group of pregnant women, despite having the highest absolute number of eligible women, had a lower enrollment rate (64.5%) than the other groups, even though they were aware of the non-monetary recognition offered for participation. The existing evidence on this matter is not conclusive. While some studies highlight that the presence of incentives, often referring to monetary or material support, plays a crucial role in encouraging participation among low-income populations, others have observed that merely mentioning such benefits can be a decisive factor in a pregnant woman's decision to participate [35]. However, the lack of permission from family members, particularly spouses, may significantly influence a woman's ability to autonomously decide whether to take part in such studies, especially in certain social and cultural contexts [39].

Lack of time, which has also been mentioned in the literature as an important limitation for participation in epidemiological studies [38], was reported especially by floricultural/agricultural and non-agricultural workers. Women in these two groups have an average workday of 40 h, with extended ranges up to 100 h. In these cases, moreover, the fact that the study data collection process required the use of varied and extensive instruments, at various times over a period of two to three years, may have influenced the decision of these women to participate, especially when their reproductive and childcare role competes with their work schedule. This may have been the case for floricultural/agricultural workers, who had a higher number of living children (1.3) compared to the other two groups (approximately 1 for each group). However, the enrollment rate of women in these groups was relatively high, 74% for floricultural/agricultural workers and 81% for non-agricultural workers, may reflect the importance that the study could have for them, considering the history of reproductive health complications among women working in flower farms in the Cayambe-Pedro Moncayo area [12, 37]. Some of the non-agricultural workers may well have been engaged in agriculture at some point in their lives, especially considering that rural women are more likely than men to be engaged in seasonal agricultural work [24]. A higher rate of participation in pregnant women in these two working groups compared to the non-worker group could reflect a higher risk perception in the former, a trend observed in studies of environmental and occupational exposures [38].

Finally, one issue observed during the recruitment process across all groups was the concern about venous blood sampling from the newborn within two weeks of birth. This concern, often tied to avoiding pain or harm to the baby, was expressed by some eligible participants and their partners when reviewing the informed consent. Although venipuncture is considered the method of choice for blood sampling in term neonates and is reported to be less painful than capillary heel prick collection [40, 41], the perception of it being more invasive acted as a deterrent for some. In the context of the SEMILLA study, venous blood sampling was required to obtain sufficient volume and analytical precision for thyroid hormone analysis, a key biomarker of interest. While less invasive alternatives, such as capillary sampling, were considered, they did not meet the requirements for volume and laboratory accuracy. In this regard, Galea and Tracy [38] note that studies requiring invasive procedures tend to have lower participation rates than those posing fewer burdens on participants. This highlights the importance of addressing participant perceptions, which, even when not clinically accurate, can significantly influence willingness to participate.

The recruitment strategies employed in this study, although diverse and contextually adapted, faced several limitations. Some approaches, such as those relying on institutional collaboration or digital outreach, may have been more effective in reaching women already connected to the formal health system or with access to social media, potentially underrepresenting more isolated or disconnected populations. Additionally, strategies that depended on institutional records, such as the Obstetric Census, were at times limited by incomplete or outdated data, which affected their reach. Reaching women living in geographically dispersed and remote rural areas posed significant logistical and financial challenges. While the study covered the cost of private transportation to facilitate participation, longer travel times for these participants required greater investment of resources and limited the number of women the team could feasibly reach, especially in the most distant communities. Although modest non-monetary recognition was used to encourage collaboration from institutional and community spokespersons, this approach may not have been equally effective for all. Furthermore, a broader context of increased insecurity and structural violence in the country may have affected trust and willingness to participate, particularly among women unfamiliar with research activities.

Although a range of demographic and occupational characteristics were assessed, the possibility of unmeasured confounding factors influencing the differences observed between participant groups cannot be ruled out. One such factor may be the perception of pesticide exposure and general environmental concern. Evidence from other contexts suggests that individuals living near floricultural or agricultural zones may perceive themselves at greater risk for pesticide-related health problems, even when objective exposure is low or uncertain. For instance, Gerbers et al. [42] found that higher levels of perceived exposure and environmental worry were associated with increased reporting of health symptoms and health care use, particularly among those with greater self-perceived sensitivity to pesticides. In the context of SEMILLA, such perceptions could have affected women’s decisions to participate, either deterring them due to fear or encouraging them to join out of a desire for health screening and follow-up. Because the recruitment process deliberately avoided emphasizing pesticide exposure in order to minimize selection bias, these perceptions were not explicitly measured at the time of recruitment. This omission limits the ability to assess their potential role in shaping recruitment outcomes. Although the extent of their influence remains uncertain, such perceptions may have contributed in subtle ways to women's decisions and represent an area for further exploration in future studies.

Among the strengths of the study is the fact that it succeeded in having a high recruitment rate of pregnant women between 8 and 20 weeks of pregnancy, especially considering that lower gestational age often relates to lower participation rates [14, 37]. The use of different recruitment strategies, mentioned in other studies [34], allowed us to have a representative sample of the population of pregnant women in the area, stratified according to characteristics of maternal occupational activity, strengthening the internal validity of the study for the analysis of environmental and occupational exposures in pregnant women in the Cayambe-Pedro Moncayo area, and their relationship with the growth and neurocognitive development of their babies. Strategies such as active community outreach, direct communication, and the use of a Facebook fan page, although less effective in direct recruitment, were important and necessary for consolidating and strengthening the study’s presence and credibility within the social context of the research area. This was a crucial factor in fostering trust and promoting participant recruitment, particularly in community-based settings [14].

The recruitment process developed for the SEMILLA study provides valuable guidance for future environmental and occupational birth cohort studies that require stratified recruitment to establish comparability groups. Such stratification strengthens the research questions and study hypotheses, while also facilitating the identification and exploration of potential confounding and modifying factors.

## Conclusions

In summary, birth cohort studies that aim to analyze occupational and environmental exposures, stratifying recruitment according to comparability groups, require differentiated strategies. In the case of SEMILLA, the stratification of recruitment according to the category "maternal occupational activity" allowed for participation rates above 64% in the three groups of pregnant women. Given that the study was community-based, the most effective strategies were those that included links with institutional and community actors. Among them, the spokespersons, through non-monetary recognition, and the Obstetric Census allowed for a greater number of pregnant women to be recruited. In both cases, social positioning of the study was necessary to generate trust in the population. Liaison with the public sector in the provision of health services was important during the recruitment process, since existing regulations in the country facilitate and promote timely care in the early stages of pregnancy. Our study highlights the importance of the use of differentiated recruitment strategies that are tailored to the characteristics of the population. It is important to consider aspects such as paid work, available time and women's autonomy in order to adapt the recruitment process to the living conditions and availability of potential participants.

## Data Availability

Once the SEMILLA data have been fully collected, analyzed, and the main findings published, we will consider sharing data with qualified researchers. Given that our study population is vulnerable, particular care must be taken to protect participants’ privacy. Data sharing will be considered under a formal data-sharing agreement, ensuring compliance with ethical guidelines and institutional regulations. This agreement will require users to: (1) utilize the data strictly for predefined research purposes agreed upon in advance, (2) obtain approval from their Institutional Review Board (IRB), (3) ensure secure storage and handling of the data using appropriate technology, (4) acknowledge the data source in any publications and cite the primary investigators, (5) delete or return the data upon completion of the agreed-upon analyses. Requests for data access will be reviewed on a case-by-case basis. Researchers interested in obtaining access should contact the corresponding author for further details. Requests for data access will be reviewed on a case-by-case basis. Researchers interested in obtaining access should contact the corresponding author for further details.

## Abbreviations

IESS: *Instituto Ecuatoriano de Seguridad Social* (Ecuadorian Social Security Institute)
MSP: *Ministerio de Salud Pública* (Ecuadorian Ministry of Public Health)
SEMILLA: Study of Environmental Exposure of Mothers and Infants Impacted by Large-Scale Agriculture
TAPS: *Técnicos de Atención Primaria en Salud* (Primary Health Care Technician)
